# A Survey on Environmental Sustainability Among Anesthesiologists: An Opportunity for Changing Behaviors

**DOI:** 10.7759/cureus.53367

**Published:** 2024-02-01

**Authors:** Patrícia Santos, Beatriz Oliveira, Cristina Romão, Nuno Leiria

**Affiliations:** 1 Anesthesiology, Centro Hospitalar Universitário de Lisboa Central, Lisbon, PRT

**Keywords:** recycling, environment pollution, waste, anesthesia, environmental sustainability

## Abstract

Introduction

Environmental sustainability (ES) is a current issue related to natural resource scarcity, pollution and climate change. Although operating rooms (ORs) comprise a small proportion of the entire hospital infrastructure, they significantly impact the environment. Anesthesiologists are ideally positioned to assume leadership, mitigating this negative impact regarding OR waste in the environment. We created a Green Team, comprising multidisciplinary professionals from different areas of a tertiary Portuguese medical center, and conducted a survey that was sent to all the institution’s anesthesiologists to assess the current state of ES.

Results

From the sample of 133 participants, 101 responses were obtained. Concerning knowledge and training on ES, a significant portion of the respondents (66.7%) seem to attribute “great importance” to the subject. As to the greatest barrier to waste separation in the OR, several respondents highlighted the issues of “inadequate information/education/training” (62.6%) and “lack of support from hospital/OR in-chief/administration” (26.3%). Finally, among seven methods to raise awareness of ES, “training during residency” was the top choice for these professionals, with 52.5% of the votes.

Discussion

Most anesthesiologists who responded to the survey recognize the utmost importance of ES and have perceived the environmental impact of their anesthetic practices in the OR. Overall, this tendency is consistent with other international studies. Moreover, most of those surveyed separate waste at home and want to extend this practice -in a more structured approach- to their workplace, with an effective separation of anesthetic and general waste in the OR.

Conclusion

Professionals perceive barriers to performing green practices, whether the lack of environmental education and awareness, the absence of recycling containers or waste separation bags, or the lack of protocols and guidelines implementing these circuits. With the publication of this work, we aim to encourage other institutions to implement ES projects in their hospitals and ORs.

## Introduction

Environmental sustainability (ES) is a current issue related to natural resource scarcity, pollution, and climate change, as well as its repercussions on ecosystems. Pollutants generated in hospitals are either released into the environment without alteration or incinerated, contributing to a significant release of carbon dioxide into the atmosphere: 5.2% of global greenhouse gas (GHG) emissions in 2019 [1]. This adds to the pool of GHGs, which play a role in climate change, a phenomenon that impacts both social and environmental factors influencing health [1].

Amid health care services, hospitals play a significant role in ES. Despite representing a small portion of all hospital infrastructure, operating rooms (ORs) disproportionately contribute to the hospitals’ general environmental impact, given their high residue production. Indeed, a few studies report that the OR is responsible for about 20 to 70% of hospital waste [1,2]. It is therefore an ideal area to start this intervention toward sustainability, acting as a promoter for the remaining hospital departments and organizations.

In this instance, the anesthesiology input is important. Since one-quarter of OR residues are generated by anesthesiologists and 60% are recyclable (unless contaminated by body fluids) [3], these clinicians are ideally positioned to assume leadership, mitigating this negative impact of OR waste on the environment. 

While the overall impact of inhalational anesthetics on global climate change is minor, certain agents, such as desflurane and nitrous oxide, have a notably higher environmental impact, around 20 times greater than isoflurane and 15 times more than sevoflurane [4,5]. To mitigate the environmental footprint, anesthesiologists can adopt practices like using agents with lower environmental impact, using low-flow anesthesia, and employing techniques such as regional anesthesia and total intravenous anesthesia (TIVA) to minimize the reliance on inhalational agents [1,6,7]. It is crucial, however, to always consider these measures within the broader context of achieving optimal patient outcomes in each individual case [1]. 

According to anesthesiology societies’ current recommendations, including those of the European Society of Anesthesiology and Intensive Care [1,4], we created a Green Team, a multidisciplinary one comprising professionals from different areas of the Centro Hospitalar Universitário de Lisboa Central (CHULC), a tertiary Portuguese medical center. This Green Team must intervene to conceive a plan to remodel ORs into greener departments, and requires active involvement and collaboration across various departments including: anesthesiology, surgery, nursing, sugical technicians, facilities services, hospital administration, supply chain and health care informatics. 

Our initial main goal was to evaluate current in-hospital practices, healthcare professionals’ perceptions of ES in the OR, and the policies that could be applied to improve them. We then intended to design an action plan to reduce the OR’s carbon footprint.

We assessed ES’s status quo using a survey sent to all anesthesiologists (consultants and residents). Its purpose was to answer the following questions: (1) How many anesthesiologists consider the environmental footprint of OR activities, (2) what are the largest barriers to waste separation in the OR, and (3) what is the best method to raise environmental awareness among anesthesiologists?

## Materials and methods

Study design 

Based on literature review and available consensus and according to pre-existing international templates [8-11], a survey was conceived. It was structured to (1) determine respondents’ attitudes toward waste separation and recycling processes in the OR, (2) identify potential barriers to performing ES practices, (3) propose educational programs on ES, and (4) compare the answers obtained with those previously published. 

The questions were bundled into six sections: (A) Demographics, (B) Knowledge and Training, (C) Current Practices, (D) Barriers to Sustainability, (E) Future Efforts, and (F) Open-Ended Question (for additional comments). Comprising 34 questions, the survey included multiple choice, Likert scale (with five items: “strongly agree,” “agree,” “uncertain/neither agree nor disagree,” “disagree,” and “strongly disagree”), and open-ended questions.

Sample size

This survey was sent to all physicians of CHULC’s anesthesiology department (92 consultants and 41 residents, totaling 133 participants).

Ethical considerations

Approval was obtained from the board of directors after the study’s submission to CHULC’s research center and evaluation by its ethics committee (reference number: CES 975/2020). Each response in the survey was given without revealing the participant's identity, and their agreement to take part was inferred from their active participation.

Data collection

The survey was emailed to 92 consultants and 41 residents from anesthesiology, totaling 133 participants. It remained available for over a month, and a reminder was established one week before the closure deadline. All the information on the study was conveyed at the beginning of the survey, and informed consent was obtained. Moreover, anonymity and confidentiality was ensured. From the 133 participants came 101 responses, which were managed using Google Forms (https://docs.google.com/forms/).

Survey validity 

The survey was piloted by a small sample of three anesthesia consultants and three anesthesia trainees/residents. No changes were proposed after the feedback process was concluded.

Statistical analysis

Statistical analysis was performed using Statistical Package for Social Sciences (SPSS) version 27.0 (IBM Corp., Armonk, NY, USA).

## Results

The survey results, which were obtained using the aforementioned methodology, are presented below.

Section A: demographics

From a sample of 133 participants constituting CHULC’s anesthesiology department, 101 responses were obtained. Of the 101 respondents, 99 authorized data collection, generating a response rate of 74.4%. Table 1 summarizes the respondents’ demographic characteristics.

**Table 1:** Respondent Demographics (Section A; n = 99) HC = hospital consultant; OR = operating room

Characteristic		n	%
Sex	Female	83	83.8%
	Male	16	16.2%
Age	< 30 Years	27	27.3%
	30–49 Years	47	47.5%
	50–65 Years	24	24.2%
	> 65 Years	1	1%
Professional Category/Role	Trainee/Resident	37	37.4%
	HC	26	26.3%
	Graduate HC	30	30.3%
	Senior Graduate HC	6	6.1%
Years of Practice	1–2	15	15.2%
	3–5	21	21.2%
	6–10	20	20.2%
	11–15	13	13.1%
	16–20	3	3%
	21–30	16	16.2%
	> 30	11	11.1%
Most Frequent Workplace	OR	96	97%
	Anesthesia Outside the OR	0	0%
	Perioperative Appointment	0	0%
	Chronic Pain Appointment	2	2%
	Coordinating the Department	1	1%

Despite their years of anesthesia practice (including completed years of residency), the respondents revealed significant variety. About 41.4% have three to 10 years of professional experience. Furthermore, most respondents (97%) work in the OR, which supports the analysis in this hospital sector.

Section B: knowledge and training

Regarding the Knowledge and Training on ES section, a significant portion of the respondents (66.7%) seem to attribute “great importance” to the subject, with most (98%) acknowledging its magnitude (Table 2). Almost 70% of the respondents “strongly agree” on the environmental impact of products and practices associated with OR activity, and 100% “agree” or “strongly agree” that training and education on ES in the OR are important.

**Table 2:** Results for Section B: Knowledge and Training (n = 99) ES = environmental sustainability; OR = operating room

		n	%
How important is ES?	Irrelevant	0	0%
	Not Very Important	0	0%
	Important but Not My Responsibility	2	2%
	Important	31	31.3%
	Very Important	66	66.7%
To what extent do you agree with the following statement: “The environmental impact of the products and practices associated with OR activity is an important factor to consider.”	Strongly Disagree	0	0%
	Disagree	0	0%
	Uncertain/Neither Agree nor Disagree	1	1%
	Agree	29	29.3%
	Strongly Agree	69	69.7%
To what extent do you agree with the following statement: “Training on ES in the OR is important.”	Strongly Disagree	0	0%
	Disagree	0	0%
	Uncertain/Neither Agree nor Disagree	0	0%
	Agree	38	38.4%
	Strongly Agree	61	61.6%
Have training/raising awareness activities concerning this topic been developed?	Yes	1	1%
	No	76	76.8%
	Uncertain	22	22.2%
Have you received training on ES in anesthesia?	Yes	5	5.1%
	No	94	94.9%
	Uncertain	0	0%
Is ES part of the trainees/residents’ curriculum in anesthesiology?	Yes	4	4%
	No	65	65.7%
	Uncertain	30	30.3%

Of the 99 respondents, 76.8% stated that no awareness actions or environmental training on this topic have been developed, with 94.9% indicating the absence of environmental training in anesthesia and 65.7% stating that sustainability is not addressed in the trainees/residents’ curriculum in anesthesiology, data that highlight this considerable training gap (Table 2).

Section C: current practice

Although 75% of respondents “strongly agree” that they separate domestic waste at home and 64% want to separate waste in the OR (“strongly agreeing” with this statement), only 36% “agree” that anesthetic waste is effectively separated in the OR where they usually work. Most of the respondents seem to consider the environmental impact of their actions when choosing anesthetic products, with 33% “strongly agreeing” and 30% “agreeing” with this statement (Table 3).

**Table 3:** Results for Section C: Current Practices Part I (n = 99) OR = operating room

	Strongly Disagree		Disagree		Neither Agree nor Disagree		Agree		Strongly Agree	
	n	%	n	%	n	%	n	%	n	%
I separate domestic waste at home.	1	1%	3	3%	1	1%	20	20.2%	74	74.7%
Anesthetic waste is separated in the OR where I usually work.	14	14.1%	18	18.2%	26	26.3%	35	35.4%	6	6.1%
I want to separate anesthetic waste/general waste in the OR.	1	1%	3	3%	5	5.1%	27	27.3%	63	63.6%
I consider the environmental impact of my actions when choosing anesthetic products (e.g., surgical gowns/clothing, laryngeal masks, and circuits).	1	1%	14	14.1%	22	22.2%	30	30.3%	32	32.3%

Concerning what products are separated for recycling in the OR, according to 45.5% of the respondents, “paper/cardboard” is the most commonly separated for recycling, followed by “glass,” “batteries,” and “plastic.” Nevertheless, worth noting is that, despite efforts to reduce waste production in the OR, 28.3% of respondents “don’t know” whether products are separated for recycling, and 23.2% reported that they “don’t separate” (Table 4). 

**Table 4:** Products That Are Separated for Recycling (Section C; n = 99)

	n	%
Paper/Cardboard	45	45.5%
Glass	23	23.2%
Plastic	18	18.2%
Metal	3	3%
Batteries	19	19.2%
Electronics	7	7.1%
We don’t separate.	23	23.2%
I don’t know.	28	28.3%

In an open-ended question, the respondents were asked about the reasons underlying their unawareness of recycling in the OR. In order to analyze the responses from the implemented survey, we grouped those addressing similar concerns according to their underlying theme. As a result, their responses indicated a lack of knowledge on the subject due to the absence of training and formative activities (47.1%), a lack of recycling containers or waste recycling bags in the OR (14.7%), and a lack of guidelines or protocols implemented by hospital administration (14%). However, being that some of the answers didn’t fit within any of the aforementioned categories, they were presented separately. We decided to still include them in the analysis, to ensure that even the topics or suggestions that were less commonly presented, would still be fairly addressed in our evaluation. This is the reason for some of them having been presented as direct quotes (Table 5).

**Table 5:** Causes of Lack of Knowledge Related to Recycling Initiatives in the OR (Section C; n = 34) OR = operating room

	n	%
Lack of Knowledge/Information/Training/Awareness	16	47.1%
Lack of Recycling Containers for Residue Separation	5	14.7%
Lack of Organization/Guidelines or Protocols Implemented by Hospital Administration	5	14.7%
Inertia/Unwillingness/Lack of Incentive	3	9%
Economic Impact	1	2.9%
“The issue lies within the domain of facility services”	1	2.9%
“The OR does not comply for recycling processes”	1	2.9%
“It is difficult change habits and circuits”	1	2.9%
“We already did a waste separation trial, but everything stayed the same”	1	2.9%

In addition, some improvement proposals, such as “regular training and raising awareness activities” (39.5%), “the need for separate containers in different colors for different materials” (30.2%), and “legal imposition through implementing institutional protocols with appropriate circuits for recycling” (30.2%), were suggested (Table 6).

**Table 6:** Suggestions from the Survey Respondents to Change Environmental Practices in the OR (Section C; n = 43) OR = operating room

	n	%
Regular Training/Awareness/Information	17	39.5%
Conditions for Waste Division (Containers)	13	30.2%
Institution of Guidelines/Protocols/Adequate Circuits	13	30.2%

While 89.9% of the respondents most frequently use sevoflurane as an inhaled anesthetic agent, only 46.5% stated that the environmental impact of inhaled anesthetic agents influences this choice. Moreover, 34.3% do not consider the environment when choosing anesthesia, and nearly 20% are unaware of these agents’ potential environmental impact (Table 7). 

**Table 7:** Results for Section C: Current Practices Part II (n = 99)

		n	%
Which inhaled anesthetic agent do you use most frequently?	Desflurane	10	10.1%
	Sevoflurane	89	89.9%
Does the environmental impact (e.g., global warming and ozone depletion potential) of inhaled anesthetic agents affect your choice of anesthesia?	Yes	46	46.5%
	No	34	34.3%
	I am unaware of the environmental impact of such agents.	19	19.2%
What items should be placed in the white waste bags of Group III biological hospital waste?	Anything That Has Come Into Contact With the Patient	37	37.4%
	Anything Contaminated With Blood or Other Bodily Fluids	59	59.6%
	Anything Visibly Dripping or Soaked in Blood or Other Bodily Fluids	3	3%
Do you use prefilled syringes?	Yes	17	17.2%
	No	82	82.8%
Does the purchasing department consider their environmental impact when purchasing anesthetic agents or selecting products (e.g., laryngeal masks and circuits)?	Yes	2	2%
	No	12	12.1%
	Uncertain	85	85.9%
Does your hospital have a sustainability task force?	Yes	0	0%
	No	21	21.2%
	Uncertain	78	78.8%

Regarding the adequate management of waste in the OR, the respondents were asked about the items to be placed in the white waste bags of Group III biological hospital waste, and although 59.6% of respondents mentioned placing “anything contaminated with blood or other bodily fluids” in these bags, 37.4% reported placing “anything that has come into contact with the patient” in them, a practice that does not correspond to correct hospital guidelines (Table 7).

Regarding “choosing anesthesia without inhaled anesthetic agents,” 52.5% of the respondents have considered using total intravenous or regional anesthesia. Furthermore, 33% mentioned “using reusable products instead of single-use/disposable products.” However, compared to concerns about electrical expenses (e.g., “turning off lights”), “using reusable sharps disposal containers,” “separating waste,” “using reusable products,” and even “reprocessing single-use medical equipment” are less implemented practices by the respondents (Table 8).

**Table 8:** Practices You Perform Considering ES in the OR Where You Usually Work (Section C; n = 99) ES = environmental sustainability; OR = operating room

	n	%
Choosing Inhaled Anesthetic Agents Based on Ecological Footprint	51	51.5%
Choosing Anesthesia Without Inhaled Anesthetic Agent	52	52.5%
Using Prefilled Syringes	11	11.1%
Using Reusable Products (e.g., Surgical Gowns, Laryngeal Masks, and Anesthetic Circuits) Instead of Single-Use/Disposable Products	33	33.3%
Separating Waste	36	36.4%
Using Reusable Sharps Disposal Containers	48	48.5%
Turning off the Anesthesia Machine and Other Equipment in the OR After Use	82	82.8%
Turning off Lights When Leaving Empty Common Areas	69	69.7%
Reprocessing Single-Use Medical Equipment (e.g., Laparoscopic Instruments, Pulse Oximeters, and Laryngeal Masks)	23	23.2%
Donating Unused Medical Equipment to Medical Missions	17	17.2%
Engaging the Industry to Promote Greener Practices	7	7.1%
Preferring Unbleached Paper and Double-Sided Printing	48	48.5%
I am unaware of these efforts.	5	5.1%

Section D: barriers to sustainability

In a single-choice format, the respondents were asked about the greatest barrier to waste separation in the OR (Table 9). Several highlighted “inadequate information/education/training” (62.6%) and “a lack of support from hospital/OR in-chief/administration” (26.3%).

**Table 9:** What Do You Consider the Greatest Barrier to Waste Separation in the OR? (Section D; n = 99) OR = operating room

	n	%
Staff Attitude	5	5.1%
Cost	2	2%
Inadequate Information/Education/Training	62	62.6%
Safety	0	0%
Time	2	2%
Lack of Space for Waste Separation/Recycling Facilities	2	2%
Lack of Support From Hospital/OR In-Chief/Administration	26	26.3%

Concerning the actions the respondents are willing to take to increase ES in the OR, they revealed a relevant level of commitment to these efforts, with 87.9% willing to “change behaviors,” 78.8% willing to “spend time self-educating,” and 65.7% willing to “provide time to train others” (Table 10). 

**Table 10:** Which of the Following Actions Are You Willing to Take to Increase ES in the OR? (Section D; n = 99) ES = environmental sustainability; OR = operating room

	n	%
Provide Time to Train Others	65	65.7%
Spend Time Self-Educating	78	78.8%
Change Behaviors	87	87.9%
None of the Above	2	2%

Section E: future efforts

Several respondents stated that no “training/raising awareness activities” regarding ES in anesthesiology have been developed, confirming a significant lack of knowledge on the subject. While more than half of the respondents (56.6%) seem “committed” to seeking more information on ES anesthesia practices, important to note is that 31.3% claimed to be “not very committed” (Table 11). In addition, among seven methods to raise awareness of ES, “training during residency” was the leading choice for these professionals, with 52.5% of the votes.

**Table 11:** Results for Section E: Future Efforts (n = 99) ES = environmental sustainability

		n	%
Rate your commitment to seeking more information on sustainable anesthesia practices.	Uncommitted	2	2%
	Not Very Committed	31	31.3%
	Committed	55	55.6%
	Very Committed	10	10.1%
What is the most effective method to raise awareness of ES among anesthesiologists?	Training During Residency	52	52.5%
	Workshops	20	20.2%
	Online	8	8.1%
	Congresses	1	1%
	Journal Clubs	3	3%
	Group Interaction/Peer Discussion	13	13.1%
	Autonomous Learning/Reading	2	2%

Section F: open-ended question

In a closing open-ended question, the respondents were allowed to freely comment on ES. From 99 respondents, 17 final comments were obtained. We analyzed these free-text comments employing a thematic methodology, leading to the identification of four themes: 1) Importance of training in ES, especially during residency; 2) Importance of implementing these measures through institutional protocols for waste separation in the OR; 3) Importance of choosing an adequate anesthetic strategy, reducing electrical and water consumption; 4) Congratulations regarding our initiative. Illustrative examples of these are presented in Table 12. 

**Table 12:** Results for Section F: Open-Ended Question (N = 17) OR = operating room

Theme	Sample Comments	n	%
Importance of Training in Environmental Sustainability	“I think, besides training during internship, training should also be available to specialists who, I am sure, will often have less correct practices due to lack of knowledge.”; “Teaching people to be more responsible about this subject and training them are key.”; “Periodically emphasizing how to proceed in the BO for environmental sustainability would be important.”	8	47.1%
Importance of Implementing these Measures through Institutional Protocols for Waste Separation in the OR	“Training and implementation of these initiatives at the level of hospital administration/”core structure” would be essential to standardize these practices.”; “It is essential to create standards and protocols on this topic.”	4	23,5%
Importance of Choosing an Adequate Anesthetic Strategy, Reducing Electrical and Water Consumption	“Adequate spending on electricity and water and Choice of anesthetic technique, are fundamental to environmental sustainability.”	2	11,8%
Congratulations Regarding our Initiative	“With this Survey I got motivated to change habits and collaborate with any initiative in this context that my hospital launches. Congratulations!”; “Current topic with great relevance, thank you.”	3	17,6%

## Discussion

Anesthesiologists must be role models in advocating for environmentally friendly causes, exhibiting a dedication to sustainability in their individual lives and professional spheres [1]. Most of the anesthesiologists who responded to the survey recognize the utmost importance of ES and have perceived the environmental impact of their anesthetic practices in the OR. This finding is consistent with those of other international studies on ES in the OR [8-11].

Most of the surveyed population separate waste at home and want this practice to extend, in a more structured approach, to their workplace, with an effective separation of anesthetic and general waste in the OR. When these data are compared with those of previously published studies, conducted in other countries [8-11], the results are similar, revealing anesthesiologists’ overall tendency toward a willingness to recycle.

However, only 41.5% “agree” or “strongly agree” that anesthetic waste is effectively separated in the OR where they usually work. Roughly a quarter of the waste generated in operating rooms is attributed to anesthesiology, with an estimated 60% of this waste being potentially recyclable. A considerable portion of this recyclable material is produced during the preparatory stages, before the patient's arrival in the operating room, thus ensuring its cleanliness and potential to be recycled [11].

Careful and proper separation of medical waste is especially important for health organizations, when considering the expenses associated with their elimination [12]. If non-infectious waste is mixed with infectious one, and thus contaminated, not only its processing becomes more expensive, but its environmental impact is also greater [13]. The cost of elimination of infected materials is five times higher when compared to non-infectious ones [14], not just because it requires higher energy processes, but also due to the fact that they have negative health and environmental effects [15]. 

It is thus vital that we educate healthcare professionals on residue recognition before their separation and elimination and provide them with the necessary tools and materials, including standardized bins and waste bags [16].

One of the biggest challenges with recyclable residues separation in the OR is the concern with contamination. Establishing recycling programs in the OR requires careful planning, as well as promoting interventions to reduce contamination of otherwise non-infectious residues. With this in mind, awareness campaigns should be developed (such as posters and pamphlets) and adequate disposal containers must be available. 

More than half of the respondents agree on the importance of training programs on this topic, as they understand that their knowledge of the environmental impact of waste and procedures related to anesthesia and activities in the OR is insufficient to guide their practice. Furthermore, several acknowledge that formative initiatives in this area are lacking, even in the curriculum of anesthesiology trainees, and should be an integral part of it. Regarding strategies to raise ES awareness, similarities between the survey results and the results of the Canadian study [8], where professionals also believed that having some training during residency was the best way to increase motivation and change behaviors, were found. 

The European Society of Anesthesiology and Intensive Care (ESAIC) Glasgow Declaration advocates that training in anesthesia should incorporate educational components focused on environmental sustainability [1]. In our hospital, the Green Team has already started training OR members with regard to this subject.

One of the most remarkable conclusions of this analysis is the gap between two realities: the awareness and importance afforded to ES and the barriers to implementing it in anesthetic practice. For instance, despite most of the respondents using sevoflurane, less than half mentioned that the environmental impact of inhaled anesthetic agents influences this choice. Some still do not consider the environment when choosing anesthesia or remain unaware of its environmental impact (Table 7). This finding leads us to confront the contradictory results in Table 3, where more than 60% of the respondents acknowledged considering the environmental impact when choosing anesthetic products (30% “agree” and 32% “strongly agree”). This may result from the lack of knowledge about the environmental impact of volatile anesthetic compounds, which emphasizes the urge to disclose strategies to reduce their ecological footprint. 

With respect to others' practices on ES, in contrast to the Canadian study results [9] -in which less than half of the sample admitted to turning off equipment and anesthetic machines in their daily practice (46.2%)- a greater fraction of our respondents (82.8%) took that into account. This option was one of the most recognisable among 13 options, which was followed by turning off the lights when leaving a common area (69.7%). Awareness associated with daily home practices can explain these concerns related to electricity consumption. 

The questions related to the hospital’s organizational commitment to implementing sustainable practices have a large percentage of “I don’t know” responses, which is concerning. A similar issue was recognized in the American study, with only 364 out of 1000 respondents being aware of the existence of a green task force at their institution [8]. This aligns with the greatest barriers to sustainability recognised in this study: with higher response rates given to inadequate information (62.6%) and lack of support from the hospital/OR in-chief/administration (26.3%). The results were similar to the Canadian study, in which the former had an answer rate of 62.8% and the latter 63.5% [10].

As such it reinforces the need for creating a team, involving leadership, focused on optimizing ES in ORs. Its main purposes should be to change practices and divulge and educate healthcare professionals in this matter. 

A global analysis revealed that all respondents acknowledged the undeniable importance of ES in modern society and its significance in hospital policies, particularly in anesthesia. Nevertheless, most admitted to having a general lack of knowledge of and professional detachment from ES. They are thus eager to undertake a substantial change toward environmental maximization in their workplace, with the introduction of awareness campaigns and ecological awareness training, including the installation of recycling bins or waste separation bags in their ORs. They therefore want to work for an organization with defined standards and protocols, promoting suitable circuits for more efficient resource management.

Following the recommendations of various anesthesiology societies (i.e., the American Society of Anesthesiologists, the European Society of Anesthesiology and Intensive Care, and the World Federation of Societies of Anesthesiologists), highlighting anesthesiologists’ prominent role -contributing to reducing the environmental impact of the activities performed in the OR- is crucial. As part of hospital sustainability initiatives, anesthesia providers should encourage and achieve quantifiable decreases in the misuse of medications, disposable equipment, and energy consumption [4].

Limitations 

This study includes only a small group of anesthesiologists from a Portuguese tertiary hospital, which might not be an accurate representation of the practices on a national scale. Furthermore, the CHULC is composed of several ORs, which are spread across different hospitals where the practice may differ. 

On the other hand, the lack of knowledge about the subject can sway the answers (e.g., the unawareness about the volatile anesthetics’ environmental impact and their different pollution profiles).

## Conclusions

This study aims to assess the CHULC’s anesthesiologists' current understanding of ES as well as their current practices. A global willingness to engage in green practices in the OR has emerged. However, professionals perceive barriers to performing these practices, whether the lack of environmental education and awareness, the absence of recycling containers or waste separation bags, or the lack of protocols and guidelines implementing these circuits. Thereby, the creation of multisectorial teams (Green Team) is the main focus of change. They should apply a plan of action, educate healthcare professionals, counter the resistance to change and idealize circuits to make recycling processes, in the OR, possible, without compromising safety.

Our Green Team has been working on environmental management initiatives in multiple ORs in our hospital. This work has resulted in the elaboration of a plan entitled “Strategic and Operational Environmental Sustainability Plan in the OR,” the goal of which is to set guidelines and promote attitudes that should reduce CHULC’s environmental impact. Our purpose is to encourage other institutions to put into action ES projects in their hospitals and ORs. 

## References

[1] Buhre W, De Robertis E, Gonzalez-Pizarro P (2023). The Glasgow declaration on sustainability in Anaesthesiology and Intensive Care. Eur J Anaesthesiol.

[2] Beloeil H, Albaladejo P (2021). Initiatives to broaden safety concerns in anaesthetic practice: the green operating room. Best Pract Res Clin Anaesthesiol.

[3] McGain E, Hendel SA, Story DA (2009). An audit of potentially recyclable waste from anaesthetic practice. Anaesth Intensive Care.

[4] White SM, Shelton CL, Gelb AW, Lawson C, McGain F, Muret J, Sherman JD (2022). Principles of environmentally-sustainable anaesthesia: a global consensus statement from the World Federation of Societies of Anaesthesiologists. Anaesthesia.

[5] Sulbaek Andersen MP, Sander SP, Nielsen OJ, Wagner DS, Sanford TJ Jr, Wallington TJ (2010). Inhalation anaesthetics and climate change. Br J Anaesth.

[6] McGain F, Ma SC, Burrell RH (2019). Why be sustainable? The Australian and New Zealand College of Anaesthetists Professional Document PS64: Statement on Environmental Sustainability in Anaesthesia and Pain Medicine Practice and its accompanying background paper. Anaesth Intensive Care.

[7] Campbell M, Pierce JM (2015). Atmospheric science, anaesthesia and the environment. BJA Educ.

[8] Ard JL Jr, Tobin K, Huncke T, Kline R, Ryan SM, Bell C (2016). A survey of the American Society of Anesthesiologists regarding environmental attitudes, knowledge, and organization. A A Case Rep.

[9] McGain F, White S, Mossenson S, Kayak E, Story D (2012). A survey of anesthesiologists' views of operating room recycling. Anesth Analg.

[10] Petre MA, Bahrey L, Levine M, van Rensburg A, Crawford M, Matava C (2019). A national survey on attitudes and barriers on recycling and environmental sustainability efforts among Canadian anesthesiologists: an opportunity for knowledge translation. Can J Anaesth.

[11] Frewen L, Grossman ES, Basson C (2022). Mostly harmless? A survey of South African anaesthetists’ knowledge and attitudes regarding environmental sustainability in the operating theatre. South African J Anaesth Analg.

[12] (2023). Waste Disposal Management. https://www.asahq.org/about-asa/governance-and-committees/asa-committees/environmental-sustainability/greening-the-operating-room/waste-disposal.

[13] Kwakye G, Brat GA, Makary MA (2011). Green surgical practices for health care. Arch Surg.

[14] Pinter M, Jardim D (2014). Segregação e diminuição de resíduos sólidos no bloco cirúrgico: uma experiência bem-sucedida. Rev SOBECC.

[15] Furukawa P, Cunha I, Pedreira M (2016). Sustentabilidade ambiental nos processos de medicação realizados na assistência de enfermagem hospitalar. Acta Paul Enferm.

[16] Kagoma YK, Stall N, Rubinstein E, Naudie D (2012). People, planet and profits: the case for greening operating rooms. CMAJ.

